# Quantitative assessment of the impact of biomedical image acquisition on the results obtained from image analysis and processing

**DOI:** 10.1186/1475-925X-13-93

**Published:** 2014-07-04

**Authors:** Robert Koprowski

**Affiliations:** 1 Department of Biomedical Computer Systems, University of Silesia, Faculty of Computer Science and Materials Science, Institute of Computer Science, ul. Będzińska 39, Sosnowiec 41-200, Poland

**Keywords:** Image processing, Expert, Operator, Measurement automation, Error, Segmentation, Ultrasound, Cosmetology, Microscope, Cornea

## Abstract

**Introduction:**

Dedicated, automatic algorithms for image analysis and processing are becoming more and more common in medical diagnosis. When creating dedicated algorithms, many factors must be taken into consideration. They are associated with selecting the appropriate algorithm parameters and taking into account the impact of data acquisition on the results obtained. An important feature of algorithms is the possibility of their use in other medical units by other operators. This problem, namely operator’s (acquisition) impact on the results obtained from image analysis and processing, has been shown on a few examples.

**Material and method:**

The analysed images were obtained from a variety of medical devices such as thermal imaging, tomography devices and those working in visible light. The objects of imaging were cellular elements, the anterior segment and fundus of the eye, postural defects and others. In total, almost 200'000 images coming from 8 different medical units were analysed. All image analysis algorithms were implemented in C and Matlab.

**Results:**

For various algorithms and methods of medical imaging, the impact of image acquisition on the results obtained is different. There are different levels of algorithm sensitivity to changes in the parameters, for example: (1) for microscope settings and the brightness assessment of cellular elements there is a difference of 8%; (2) for the thyroid ultrasound images there is a difference in marking the thyroid lobe area which results in a brightness assessment difference of 2%. The method of image acquisition in image analysis and processing also affects: (3) the accuracy of determining the temperature in the characteristic areas on the patient’s back for the thermal method - error of 31%; (4) the accuracy of finding characteristic points in photogrammetric images when evaluating postural defects – error of 11%; (5) the accuracy of performing ablative and non-ablative treatments in cosmetology - error of 18% for the nose, 10% for the cheeks, and 7% for the forehead. Similarly, when: (7) measuring the anterior eye chamber – there is an error of 20%; (8) measuring the tooth enamel thickness - error of 15%; (9) evaluating the mechanical properties of the cornea during pressure measurement - error of 47%.

**Conclusions:**

The paper presents vital, selected issues occurring when assessing the accuracy of designed automatic algorithms for image analysis and processing in bioengineering. The impact of acquisition of images on the problems arising in their analysis has been shown on selected examples. It has also been indicated to which elements of image analysis and processing special attention should be paid in their design.

## Introduction

Significant technological progress and automation in many groups of medical issues have increased an interest in full automatic measurement also in image analysis and processing [1]. This automation has been implemented so far only under specific conditions and in specific groups of medical issues. In many of them, too large inter-individual variability or the issue itself introduce the need for manual adjustments of results by a specialist. This correction usually refers to part of the analysis in which an operator has to manually enter the parameter (parameters) value or specify a point or a group of points. Physical meaning of the entered data varies and depends on the type of imaging method and analysis. The points most often indicated by an operator are related to the contour and further to the segmentation of a specific object or a group of objects [2], whereas the numerical values are related to the determination of, for example, binarization threshold and determination of weighting factors for classification. The difficulties in proposing a method of automatic image analysis are also highly dependent on other factors [3]. These include various types of imaging devices and, as a consequence, e.g. different image resolutions. Each operator also introduces additional variables, visible directly in the image, such as varying brightness or orientation of the image to which they are accustomed. This results in considerable algorithm complication. In addition, it significantly affects further steps of processing and analysis such as classification. Due to these elements, the new proposed algorithms for image analysis and processing published in many papers work correctly only for a given type of medical imaging device and only in one medical facility. It is usually the acquisition device and place which were used for testing the algorithm.

Therefore, when designing new dedicated algorithms, particular attention should be paid to:

○ the impact of experts’ work (their personal habits) on the stage of classifier training (decision tree, neural networks and others) in the developed algorithm,

○ the impact of the data acquisition method on the errors of the algorithm for image analysis and processing,

○ taking into account specific cases, patients with a very high degree of pathology and interindividual variation.

In the practical approach to this type of problems, algorithms should be appropriately modified and generalized. In this case, for the examples described below, it is assumed that the form of the algorithm is the same. This is to show what types of errors can be expected when using algorithms designed on the basis of only one group of data.

Therefore, the main aim of this study is to show the impact of image acquisition on the results obtained with dedicated algorithms for image analysis and processing proposed by the author. Some selected, specific cases of medical imaging, for which the greatest errors in algorithm operation were obtained, are presented below. The obtained erroneous results of algorithm operation are only illustrative of the problem occurring in image analysis and processing and are in no way used for testing patients.

## Material

The images were acquired from three different medical devices: a tomograph, an infrared camera and devices operating in visible light. The object of imaging were cells visible in microscopic imaging [4], the anterior segment and fundus of the eye in tomography [5], postural defects in visible light [6] and thermal imaging [7], and others. A detailed list of acquired images and the exact parameters of the acquisition devices are shown in Table 1. In total, almost 200'000 images were acquired. As for the described methods, no studies and experiments were carried out on humans. All of them were obtained during routine medical examinations and the patients were examined in accordance with the Declaration of Helsinki. The patients were adequately prepared for the tests. In particular, thermal comfort conditions were ensured and the influence of other factors on the results was eliminated. All 200'000 images were analysed using the dedicated algorithms proposed by the author which work fully automatically. The selected, most interesting cases for which the presented problems of image analysis are clearly visible are shown in this paper. The erroneous results obtained on the basis of image analysis and processing were not, in any way, used diagnostically.

**Table 1:** Summary of the performed image acquisition parameters

**Device**	**Number of images**	**Resolution [pixel × pixel]**	**Object of imaging**	**Additional comments**
NICON E 600 microscope (lens: Plan Fluor 100×/130 Oil, eyepiece 10×/22)	40	1024 × 1024	Determination of the extent of proliferation in the regenerating rat liver cells	The test material was analysed under the microscope
Systems for 3D measurement of points of the human body developed in the Department of Photogrammetry and Computer Remote Sensing WGGiIŚ AGH	800	1200 × 1600	Evaluation of postural defects	Images of selected places of the human body, signalled with Styrofoam balls-markers, were obtained using two focus free compact cameras
OCT SS-1000 CASIA, OCT Zeiss Visante OCT and SOCT Copernicus Optopol Tech. SA	100'000	256 × 1024	Evaluation of retinal layer thickness and the iridocorneal angle of the anterior segment of the eye	The images are acquired for the measuring range of 8 × 16 mm
AGEMA 470 and/or 640 AGEMA digital camera QV - 400	503	140 × 140 320 × 240 600 × 400	Evaluation of uneven temperature distribution around paraspinal areas	Patients prior to the test were stripped to the waist and stayed in a room with air temperature of 21°C, in a sitting position without any back support for about 20 minutes. Next, each of them stood with their back to the camera at a distance of 2 m from the lens
Flir SC5200 infrared camera with a photon detector with 3-5 μm spectral range,	80	320 × 256	Evaluation of the correctness of performing ablative and non-ablative treatments in cosmetology	Forcing thermal changes of the skin was done using CO_2_RE laser with a wavelength of 10600 nm; pulse mode of the laser beam emission, energy density of 4,5 J/cm2, pulse energy of 1–150 mJ, pulse frequency of 16,7 kHz, spot size - 120 μm or 150 μm.
Topcon 3D OCT-2000	25'200	884 × 512	Evaluation of Thickness loss of tooth enamel	Images were obtained in vitro by performing on 180 teeth 7 processing steps: tooth without interference, polishing with paste, etching, application of adhesive system, orthodontic bracket attachment, orthodontic bracket removal, cleaning of adhesive residue.
Corvis tonometer	13'400	200 × 576	Evaluation of the mechanical properties of the cornea and intraocular pressure	Subjects aged 17–63 were healthy (32 people including 16 women) or ill (16 people including 9 women). The ill patients had either AMD or other diseases that cause abnormal pressure in the eye

## Method

**Figure 1:**
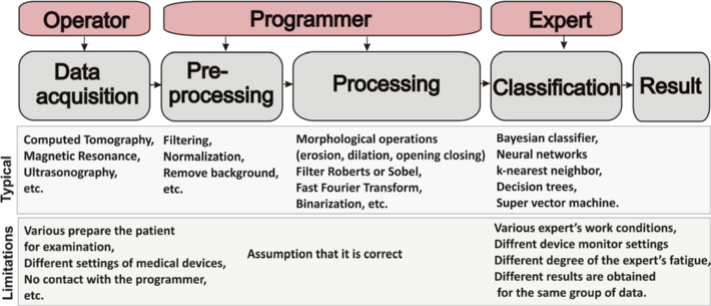
**Block diagram of the typical stages of image processing and analysis.** The block diagram shows the image acquisitions, pre-processing, proper image analysis, classification and result. Each block is affected by a lot of factors interfering measurement and, later, the results derived from a decision support system (marked in red).

A general scheme of the procedure for image analysis and processing is known from the literature [1,2]. It is the image acquisition, pre-processing, proper analysis, classification and results. The block diagram is shown in Figure 1. According to the presented block diagram, an operator is the person operating a medical imaging device who is also responsible for positioning the sample or the subject during tests. An expert is the person who identifies (indicates) characteristic areas in the image and classifies patients (for example, into two classes: "healthy", "sick") on the basis of his/her expert knowledge. A programmer is here the person who proposes a dedicated algorithm which enables automatic measurement or classification after training with a teacher (expert) [3-5]. Determination of appropriate parameters of individual blocks and possible occurrence of measurement errors are extremely interesting from a practical point of view.

The block of image acquisition by definition allows for image acquisition and its archiving. In this respect, most errors are related to the influence of different operators, different device settings and their different types (e.g., different image resolutions) on the results obtained. They include all medical imaging devices, ultrasound scanners, OCT, X-ray, microtomographic scanners, thermal imaging or magnetic resonance devices. The important factors are imaging device settings (radiation doses, acquisition time, etc.) and the method of testing (the patient in a standing, sitting, lying-down position, etc.).

The block of image pre-processing is the most resistant to any type of errors in implementation. It usually involves filtration whose aim is to remove noise and other artefacts from images. The choice of filter parameters is usually carried out on the basis of known limit values for pollution (noise) and minimum relevant object sizes. In this way, for example, the mask size of the median or adaptive filter, which is used to carry out image pre-filtration, is determined. In addition to filtering, the next most common element of pre-processing is brightness normalization, removal of all objects whose size is not within the selected thresholds and removal of illumination non-uniformity.

The block of proper image analysis is the basic processing block. The detailed form of the algorithm determines features such as versatility, optimality and reliability of the whole processing stage. Data overfitting may lead to the inability to generalize problems and thus it will be impossible to use this block in other medical facilities for other data. The elements of image analysis and processing that dominate here are morphological operations (erosion, dilation, opening, closing), filtration with Roberts or Sobel filter, fast Fourier transform, binarization with the lower or upper threshold and others.

The classification block is a typical block which uses features obtained at the image processing stage. This block is associated with classifiers such as Bayesian classifier, neural networks [8], k-nearest neighbours, decision trees or recently popular SVM (super vector machine) [9,10]. These classifiers can be trained without or with supervision. In the latter case, they are trained with a teacher – an expert in a particular field of medicine. There are often problems with the differences between the results obtained by different experts or with repeatability of one expert [11]. Another problem is the appropriate selection of the classifier and its structure (e.g., the number of layers in the case of neural networks).

The practical implementation of individual blocks influences the results obtained to a great extent. This is also the reason for the occurrence of various kinds of problems in practical implementation and creation of classifiers. For each research problem discussed below, a dedicated, automatic algorithm, implemented in Matlab and C, has been proposed in accordance with the described block diagram.

### Verification of the results obtained

One of the most significant elements of the developed image analysis software is the need to verify the results obtained or to verify the process of training a classifier with a teacher (Figure 1). This is usually an expert who, in this case, acts as a physician skilled in a particular field of medicine. Their experience and practice is used to separate healthy subjects from patients relying on other more accurate measurement methods (than the ones resulting from image analysis) and create on this basis a learning group. The results obtained by an operator are also used in assessing the accuracy of the created image analysis method, for example, in segmentation of objects or contour detection. In this case, the task of an expert is to indicate a contour, point or group of points. Expert’s participation in tests is also very important because of the subsequent evaluation of algorithm errors. Verification of the results obtained by an expert is relatively simple and well known. In the case of two classes, namely healthy and sick, it involves a comparison of the results obtained. The values which are most often calculated are false positive, false negative, true positive and true negative. On this basis, sensitivity, specificity, the ROC curve (Receiver Operating Characteristics) and AUC (area under the curve) are calculated. However, these indicators only relate to the assessment of a group of patients (a group of data). In the case of single images (accuracy of outlining the object's contour or indicating the object’s centre), the measurement error is most often evaluated in a classical way.

In this case, the specific error definition is used, i.e.:

(1) $$\delta_{w}=\frac{w_{M}-w_{P}}{w_{K}}\;100\;\left[\%\right]$$

where: *w*_*M*_ – measured value,

*w*_*P*_ – correct value,

*w*_*K*_ - conventional value, usually taken as the correct value *w*_*P.*_

A measurement error of any value is understood here as the difference between the value indicated by the algorithm and the correct value. The correct value refers here to: (1) the measurement result obtained with more accurate methods; (2) the arithmetic mean of the results of a series of measurements (3) the value calculated on the basis of theoretical premises, or; (4) the result of the expert’s work. This error is related to the conventional value, usually taken as the correct one. Unfortunately, in the case of experts, the results of their work are different, even though they always work properly. Different results are also obtained for one expert who, after intervals of several days, analyses the same material. Moreover, the work of the operator of a given image acquisition device influences the result in many different ways. These issues are discussed in the following sections.

### Different experts – different results from image processing

Correct and reproducible work of an expert in routine medical diagnosis is not always related to receiving repeatable results from image analysis and processing. It is closely dependent on the algorithm for image analysis and processing. In many practical aspects in the field of image analysis and processing, there is the afore-mentioned problem with repeatability of the expert’s work. For various expert’s work conditions, such as device monitor settings or a different degree of the expert’s fatigue, different results are obtained for the same group of data. These difficulties occur, for example, during edge detection or image acquisition performed by various experts. The results obtained by experts in image analysis are extremely important as they constitute the basis for machine learning (e.g. of a classifier) and verification of its operation.

### Example 1 - different experts – microscopic images

**Figure 2:**
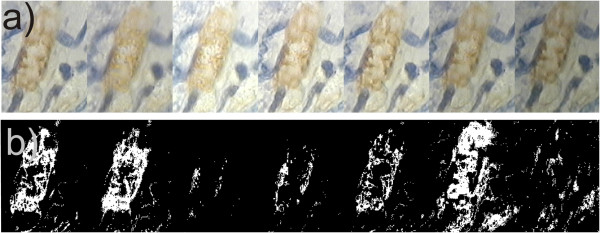
**Fragment of the same area of the formulation from the microscope for seven different experienced operators.** In their opinion, they set the microscope the best - **a)**. For each image, the algorithm for image analysis and processing must be modified in such a way so that it works properly – e.g. binarization **b)**. This results in significant technical complications. Despite this, due to the various best settings, each of the experts gets different results. The sequence of images is random.

**Figure 3:**
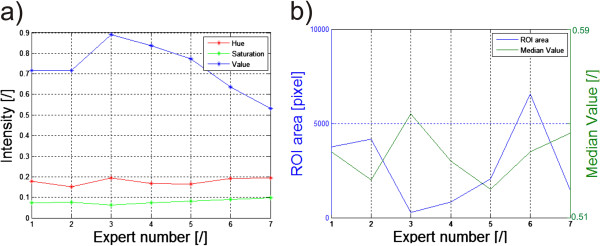
**Graphs of brightness changes for the HSV model, the surface areas and the intensity for 7 experts.** The results obtained relate to the images shown in Figure 2. The graph **a)** shows the mean values in the ROI of the components Hue, Saturation and Value for the subsequent experts. The graph **b)** shows changes in the surface area (number of pixels in the ROI) and the average intensity of the component Value in the ROI for subsequent experts.

For example, an important element in biological diagnosis is to obtain repeatable and reliable measurements derived from the afore-mentioned microscopic images of cellular structures [12-17]. For full objectification of microscopic evaluation of histological specimens and effective research documentation, their automatic analysis is increasingly being used. The key element in a series of repeated morphological measurements of cells is automatic image segmentation necessary for determining the degree of colour reaction. The parameters of microscope settings, such as specimen lighting intensity, significantly affect image quality, and thus indirectly disqualify the possibility of establishing permanent thresholds, e.g. in the process of binarization [17]. Additionally, these settings are individual for each microscope operator, even for the same sample. Figure 2 shows a fragment of a microscopic image for the settings which, according to various experts, are the best ones. Different brightness and depth of field are clearly visible. They arise from different experts’ habits and work styles. In visual assessment, in routine testing, the same repeatable results are obtained by each expert. The differences occur only during image analysis and processing with the dedicated algorithm. Unfortunately, these settings affect the results obtained from image analysis by introducing measurement errors of the surface area and the measured degree of colour reaction (in HSV model - Hue, Saturation and Value). The results obtained for simple binarization for the constant threshold *p*_*r*_ = 0.5 (for the brightness in the range from 0 to 1) for the V component are shown in Figure 2b), whereas the measurement results are shown in Figure 3a) and b). The designated maximum intensity differences for the components H and S do not exceed 0.015 while for the component V (Value), these differences are at the level of 0.2 (20%). As a consequence, the number of pixels of the separated object changes from about 200 (minimum) to 7000 pixels (nearly 140-fold increase). This causes the difference in the evaluation of the specimen colour reaction from 0.52 to 0.56 (Figure 3b).

### Example 2 - different experts – ultrasound images

**Figure 4:**
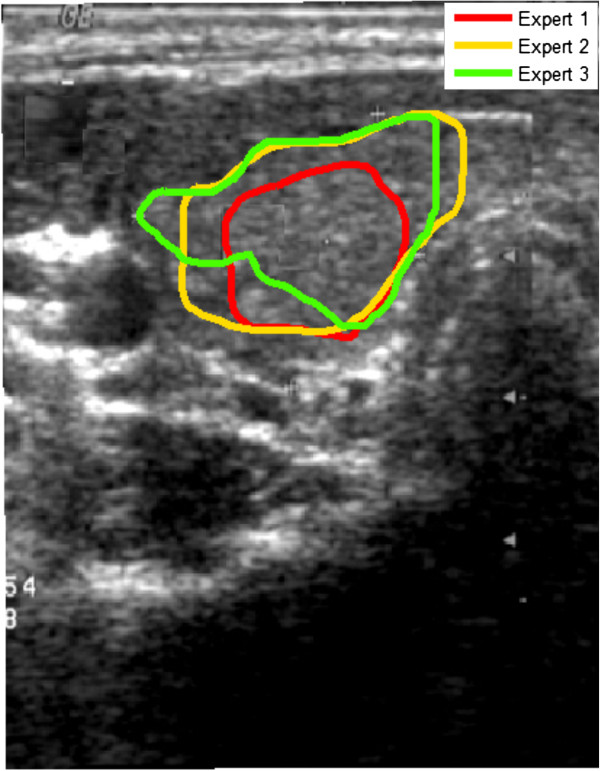
**Ultrasound image of the cross section of the thyroid.** The thyroid lobe areas marked by 3 experts are highlighted in colours. The visible differences are due to different approaches to the thyroid lobe, an attempt to eliminate shadows and other artefacts. According to some experts, shadows and other artefacts are characteristic of the thyroid lobe and should not be eliminated from the calculation.

Another example are the thyroid ultrasound images for which the thyroid lobe area must be marked [18-24]. Figure 4 shows the results of marking the thyroid area by different experts. The visible differences lead to the existence of disparities in almost all parameters calculated on the basis of the selected ROI. For example, different surface areas, different values for the maximum, minimum and average brightness and different standard deviations of the mean are obtained - Table 2. In this case, the reason for the differences in experts’ work is the way they eliminated the shadows and artefacts visible in the ultrasound image from the thyroid lobe area. The maximum values which are given in Table 2 for 3 experts are the same, whereas the differences exist in the minimum values, median values and standard deviations of the mean (std). The largest differences are for the calculated minimum value for which the difference between expert 1 and 3 is 60% for this example of the thyroid image. For the median value this is the difference of 2%. The difference of 2% in the calculation of the brightness median value is negligibly small in comparison with other error sources [18]. However, in the case of the minimum and/or maximum brightness, the algorithm for image analysis should be appropriately modified. Such modification may involve, for example, the process of brightness normalization in the ROI indicated by an expert.

**Table 2:** Results of pixel brightness measurements in the ROI for different experts

	**Intensity**			
	**Min**	**Max**	**Median**	**Std**
**Expert 1**	0.168	0.807	0.384	0.073
**Expert 2**	0.105	0.807	0.368	0.076
**Expert 3**	0.105	0.807	0.368	0.073

In summary, in each case of verifying the algorithm operation, there must be some indications that experts should follow. They should be given for any proposed new automatic algorithm for image analysis and processing. In controversial cases, extreme results obtained by experts should always be taken into account and they should indicate the accuracy of the algorithm operation.

### Operator’s influence on the results

Image acquisition is usually performed by a technician, whereas in special cases related to formal requirements by a doctor. Let us suppose further that this is a technician with the appropriate qualifications and licenses, trained to perform measurements on patients. Technicians performing image acquisition are usually directed by their own habits and developed measurement technique - using the relevant medical procedures all the time. In very rare cases, they are in contact with the person preparing the algorithm for image analysis and processing. This often leads to a situation in which the developed software must take into account the operators’ habits and their specific work. This in turn leads to an increase in the computational complexity of the algorithm, sometimes double compared to its basic part. It should be emphasized that in terms of medical procedure and metrology, the measurement is performed correctly in each case. Such problems are further shown on selected cases.

### Example 1 – the impact of image acquisition on the results – patient’s position

**Figure 5:**
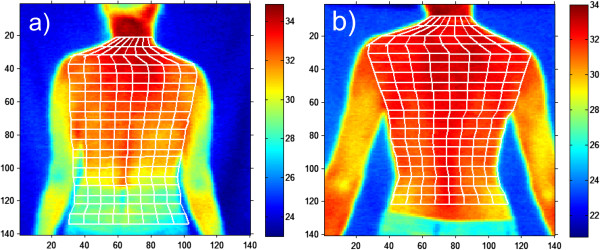
**Thermal images of the patient’s back.** The patient positioned incorrectly on the left **(a)** and properly on the right **(b)**. Correct positioning of the patient determines, to a very large extent, computational complexity of the algorithm for image analysis and processing. In this case, slight arms spread causes considerable convenience of detection of the outer contour of the patient and automatic imposition of the grid as shown in the correct image **b)**.

**Figure 6:**
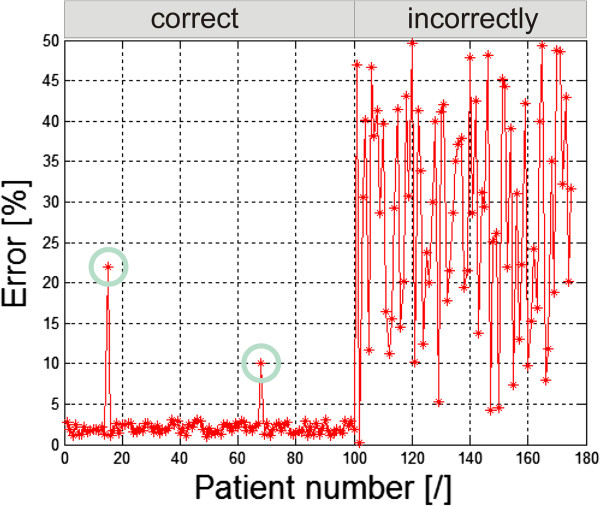
**Graph of temperature measurement errors for the extreme ROIs.** The first 100 patients had their arms spread correctly. 75 patients’ arms were relaxed and touched the hips. Green circles indicate the algorithm error. For the first 100 patients the constant error value (approximately 2%) is related to the low image resolution (140 × 140 pixels).

The first example is the situation in which an operator positioned the patient in the classical way (hands relaxed) prior to a thermal imaging test [7,25-27]– Figure 5. It resulted in problems with the correct hand contour detection and proper calculation of its temperature. This was due to the difficulty in separating the hand from the patient’s body by the algorithm. Algorithmic correction of this error is extremely difficult in this case, because it leads to finding the contour in the area where there is virtually no temperature gradient. This is only possible using anthropometric data, but at the cost of extending the computational complexity of the algorithm. In the presented case of thermal analysis, such cases occurred in 15% of the total number (503) of the patients. Figure 6 shows the graph of temperature measurement errors for the extremely localized ROIs in 100 patients for whom the measurement was carried out properly and the other 75 patients who did not adequately spread their hands. The graph (Figure 6) for the first 100 patients (for whom the measurement was carried out properly) the error is in the range of 2.3%. This is due to errors of the spatial resolution of the image - only 140 × 140 pixels. Two larger values of approximately 23% and 10% (marked with a green circle) are the algorithm errors. For the next 75 patients, due to improper patient’s positioning, the error in temperature measurement reached 31% (50% maximum). This means that such tests are entirely useless in practice. This is caused by erroneous operation of the algorithm - difficulties in the automatic correction of the contour which is hard to detect.

### Example 2 – the impact of image acquisition on the results - illumination

**Figure 7:**
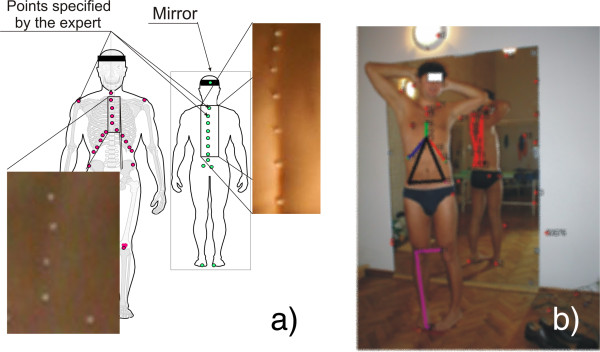
**Evaluation of postural defect using photogrammetric method.** The patient has markers (arranged by the expert) in specific points on the skin. On the basis of two images at different angles (stereovision), three-dimensional reconstruction of the position of the markers is carried out. The shadows, arising from improper lighting, generate shadows and errors in the analysis. The points marked in Figure **a)** in red are in front of the patient while the ones in green are a reflection in the mirror. In Figure **b)** the colours indicate the results of the correct operation of the algorithm for image analysis and processing involving identification (with successive colours) of the group of points assigned to specific organs.

**Figure 8:**
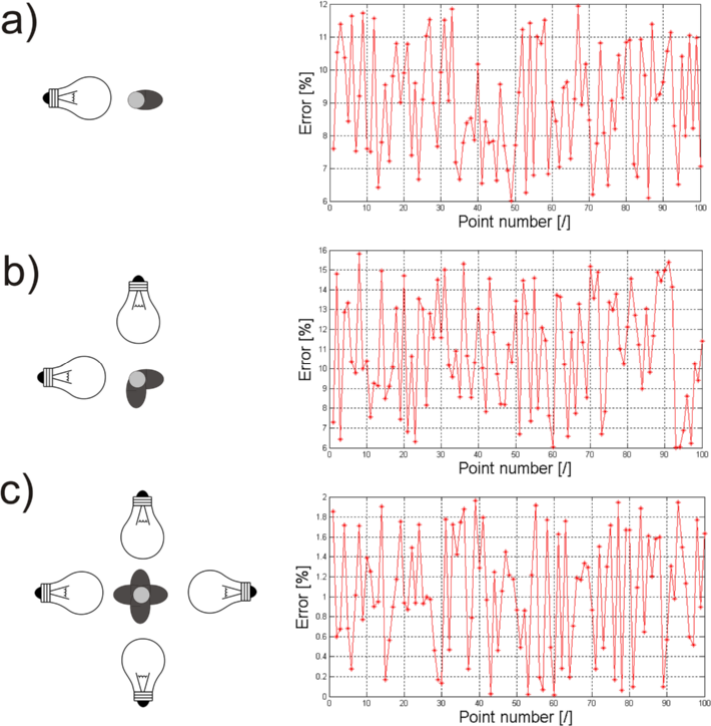
**Graphs of measurement errors for different lighting of markers.** The error values result from various types of lighting: **a)** for a single light source, **b)** for two sources, **c)** for four sources. Depending on the number of light sources, different error values are obtained. Simple correction of shifts of the places where individual points are detected is not possible here because the number and arrangement of light sources are not generally known.

Another example of operator’s individual settings and their impact on the results obtained is the way of placing lighting in the evaluation of postural defects using the photogrammetric method [6,28-30]. A crucial element here is accurate determination of the positions of the individual points of the markers on the patient’s body - Figure 7. These markers are further assigned to specific points on the skeleton and the postural defect is assessed on their basis. Their position detection in three-dimensional space is essential here. The shadows caused by improper lighting result in the measurement error of their location in relation to the results obtained for the light source placed in the camera axis. The error values are different for different types of lighting. Figure 8 shows the error values for 100 markers and one source - about 9% (Figure 8a), for two sources - about 11% (Figure 8b) and for four light sources - about 1% (Figure 8c). Automatic correction (shifts in the right axis) is not possible here due to the difficulty in determining the number of light sources and their angles of incidence in the room in which a photogrammetric system works.

### Example 3 – the operator’s impact on the results – laser treatments

**Figure 9:**
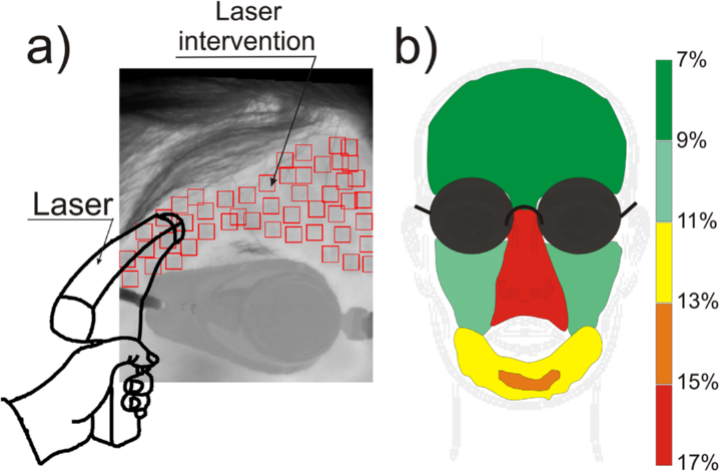
**Intermediate results obtained using the algorithm that automatically recognizes the ROI and skin areas subjected to laser intervention.** The automatically recognized locations of the ROIs enable to evaluate, even qualitatively, the accuracy of laser head moving by the operator. Gaps between the ROIs and laser radiation doses applied in several places are visible - **a)**. Figure **b)** shows coverage errors (omitted areas of the skin), and the dependence of this error on the location of the area on the face.

An interesting group of problems, as mentioned in [31-37], is the uniformity of moving the laser head while performing ablative and non-ablative treatments. Uneven moving of the laser head causes errors of overlapping radiation doses and the formation of voids with no laser interference - Figure 9a). The first error is dangerous for the patient because the patient receives a double dose of radiation - its mean value and deviation are 1.97 ± 1.5%. The other error is not dangerous for the patient but it results in the lack of uniformity of doses and thus leads to skin surface deformation; its mean value and deviation are 17.87 ± 10.5%. However, these errors are different for different locations on the patient's face. Figure 9b) shows the distribution of different error values dependent on the location of areas on the face. For the nose area the highest error values are around 16, 17%, and for the area of the cheeks they are 10%. Different values of errors result from the difference in the shapes of these areas (of the nose, cheeks, forehead). This greatly affects the comfort of moving the laser head by the operator during the treatment. In the case of the nose, difficulties in handling the head directly translate into a significant (more than double) increase in the coverage error values. Therefore, the operator must pay more attention to the areas of the face of a complex shape (e.g. nose, ear).

### Example 4 – the impact of image acquisition on the results – lack of full visibility of the anterior segment of the eye

**Figure 10:**
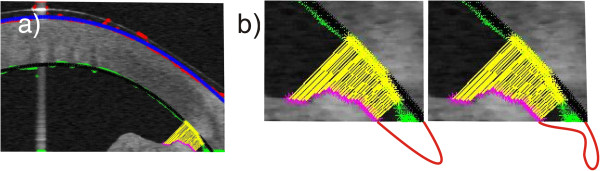
**Tomographic image of the anterior segment of the eye.** In part **a)** the colours indicate the outer and inner boundary of the cornea determined directly from the algorithm (red and green) and after approximation (blue and black). The automatic measurement of the iridocorneal angle is highlighted in yellow. Part **b)** shows in the bottom possible invisible shapes of the iridocorneal angle which are zoomed.

The last example are the problems occurring in tomographic images (B-scans) of the anterior segment of the eye [38-41]. Often, the problems are associated with the need to detect contours in places where ROIs are not fully visible. Such situations occur, for example, in tomographic images of the anterior segment of the eye - the lack of a fully visible iridocorneal angle. For this type of images the created algorithm enables automatic measurement of the iridocorneal angle. The lack of full visibility of the angle apex hinders the correct algorithm operation. Such situations occur in retrospective analysis for which tomographic images were made correctly and the evaluation of the iridocorneal angle was not the main subject of diagnosis. For these cases when the angle apex is not visible, the measurement error varies from 0 to about 20%. The exact error value is dependent on the amount of data available and the type and degree of disease which is not apparent. An example of this type of situation is illustrated in Figure 10a) and b). Figure 10b) shows possible endings of the iridocorneal angle with the red line. Due to the lack of visibility of the full angle, it is difficult to assess the degree of pathology and even roughly estimate the measurement error. These types of cases are excluded from further analysis.

In summary, for each test the contact of the operator with the algorithm designer is indispensable. The biggest problems, and thus difficulties, in proposing an algorithm occur in the case of images which are analysed retrospectively. Then, the complexity of the algorithm increases significantly for the reasons mentioned above. The measurement error also increases due to the difficulties in proposing a fully automatic algorithm for image analysis and processing. In particular, the one which would work properly without complete information (e.g., lack of a part of the image, contour discontinuity, etc.).

### Special cases

Special cases include a group of images which dedicated methods of image analysis and processing cannot handle. These are, for example, the contours of the relevant organs that are either poorly recognizable or not present at all. This information is very important, for example, for machine learning under supervision. In such cases, the patterns of typical procedures fail:

• the use of expert’s knowledge - in this case, the expert refuses, for example, to indicate the contour, because there is no additional evidence (even the outline of the object is not visible),

• the use of anthropometric information - the method fails in many cases of internal organs and their localization due to the large inter-individual variability,

• the use of the knowledge from the learning group- as in the previous case this method fails due to the large inter-individual variability and possible pathologies. This method can be used in the case of a very large learning group or small individual variability of patients (analysed images).

The only possibility is rough approximation of the searched object and using the data from previous studies of the same patient (if they exist). Previous studies also refer to, for example, previous B-scans from CT imaging. The knowledge derived from the previous or next B-scan can be taken into account in the case of three-dimensional imaging. Such situations occur repeatedly in creating algorithms for detecting contours of layers on a tomographic image of the eye fundus or contours forming the border separating two joined cells visible under the microscope. For example, such situations take place for tomographic images of teeth and the anterior segment and fundus of the eye.

### Example 1 – lack of full visibility of the enamel boundary

**Figure 11:**
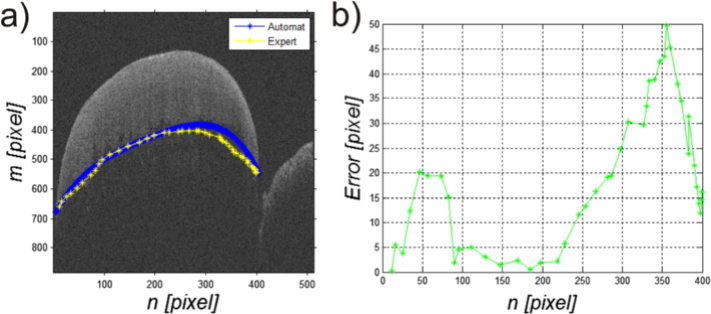
**The next steps of detecting the enamel boundary in tomographic images of teeth.** Figure **a)** shows contours of the enamel boundary marked by an expert and automatically. Figure **b)** shows the graph of errors for each image column as the relative difference between manual indication of the enamel boundary by an expert and the result of the algorithm automatic operation.

An example is the analysis made for 25'200 B-scans of 180 teeth studied in vitro [42-44] using a tomograph with automatic determination of the enamel boundary contour. In these cases, manual indication of the enamel boundary on a single B-scan by an expert is only possible in selected cases. These are the cases for which the enamel boundaries are visible (even in fragments) after image processing. For the other cases, the previous and next B-scans are taken into account. In cases where the enamel boundary is not visible in any of the B-scans, the expert has also difficulty in indicating it manually. Then, information derived from other types of imaging of the same object, e.g. grinding in the case of teeth, is used. For the discussed studies, automatic and manual methods were used (manual indication of the enamel inner layer contour by the operator/expert dentist) - Figure 11a). The error in determining the enamel boundary contour calculated on this basis did not exceed 15%. Changes in the value of the error for each pixel of an exemplary B-scan are shown in Figure 11b). It is greatly affected by the tomograph resolution (B-scan resolution) and the size of the tooth (enamel) on the stage. The maximum error values occur for the column *n* = 355 pixels and are 50 pixels. The largest correspondence between manual contour designation (by an expert) and automatic one was obtained for the starting interval *n*∈(10,25) and *n*∈(90,215) pixels.

### Example 2 – lack of visibility of all retina layers

**Figure 12:**
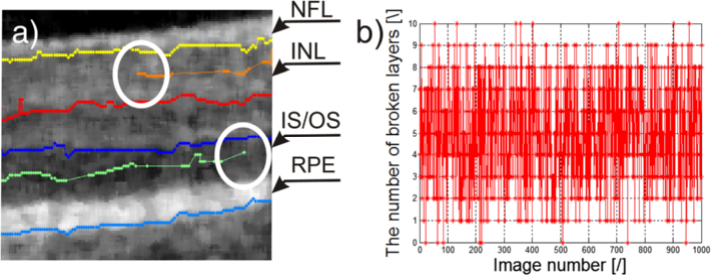
**Eye fundus image and the graph of the number of broken layers.** Image **a)** shows the boundary endings, marked white a circle. Their further indication is not possible due to the lack of information from the image and it is not anatomically present. Image **b)** shows the graph of the number of incorrect boundary endings for 1'000 sample images. The acronyms stand for: NFL-nerve fiber layer, INL-inner nuclear layer, IS/OS-inner segment/outer segment junction, RPE-retinal pigment epithelium.

Similar problems exist for tomographic images (B-scans) of the eye fundus [5,45-49]. These problems are associated with the necessity of detecting contours in places where there is not any difference in brightness. Consequently, it leads to discontinuity of individual boundaries, which is not anatomically correct. The graph of the number of incorrect boundary endings for 1'000 images and an example of their two endings are shown in Figure 12a) and b). The graph (Figure 12b) shows the number of incorrect endings of the detected layers which changes in the range from 0 to 8. For the value of 0, all layers are properly detected. For the value of 8, 8 or more layers are incorrectly ended. The value of 8 is the upper limit of the algorithm. In the illustrated graph, it can be noted that only in 168 cases the algorithm correctly detected all the layers in the full range, which represents 17% with respect to the whole analysed group. Due to incorrect operation of the algorithm in this respect, the results are not in any way used diagnostically. After the automatic correction [5] measurement error decreased to 5%.

### Example 3 – lack of information about the lens orientation

**Figure 13:**
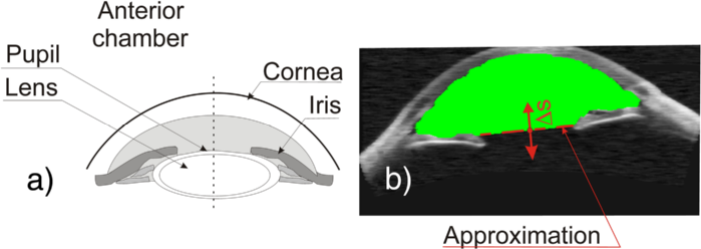
**Determination of the anterior chamber surface.** The image showing methodology of determining the anterior chamber surface – **a)**. The practical result of the algorithm - **b)**. Approximation of the lens in the case of its absence is marked in red. The area of the calculated anterior chamber surface is marked in green. Possible shift of the lens boundary is designated as *Δs*.

For the anterior segment of the eye and the previously mentioned tomographic images (B-scans), a substantial problem exists when calculating the anterior chamber volume or its surface. The anterior chamber surface must be often determined on the basis of one B-scan for a patient whose lens was removed during surgery or is not visible. Then, its approximation (most often with a straight line) is indispensable for the methods of image analysis and processing- Figure 13. Depending on the position of the line which closes the anterior chamber surface, different values of measurement errors are obtained. The measurement error in that case is referred to the results from the algorithm machine learning on several thousand other cases of patients with clearly visible lenses. Table 3 shows the obtained errors for various changes in the position of the line approximating the lens (Figure 13- the red dashed line) in the range of *Δs* ± 8 pixels. For the present example, the surface area measurement error varied from 0 to 14% when changing the position of the line approximating the lens in the range of *Δs* ± 8 pixels. For the algorithm method, the measurement error was 5%. When the line of the approximated lens contour was shifted by *Δs* = 4 pixels, the error decreased to 0%. This implies, in this case, the need for the constant correction of the algorithm operation to minimize the error in calculations. On the other hand, proper placement of the line which approximates the lens shape is quite difficult for the expert.

**Table 3:** The results of measurement of the anterior chamber volume depending on the change in the position of the line approximating the lens

***Δs *** **[pixel]**	-8	-7	-6	-5	-4	-3	-2	-1	0	1	2	3	4	5	6	7	8
**Error [%]**	14	13	12	10	8	7	6	6	5	4	3	2	0	1	2	4	5

### Example 4 – lack of full visibility of the corneal contour

**Figure 14:**
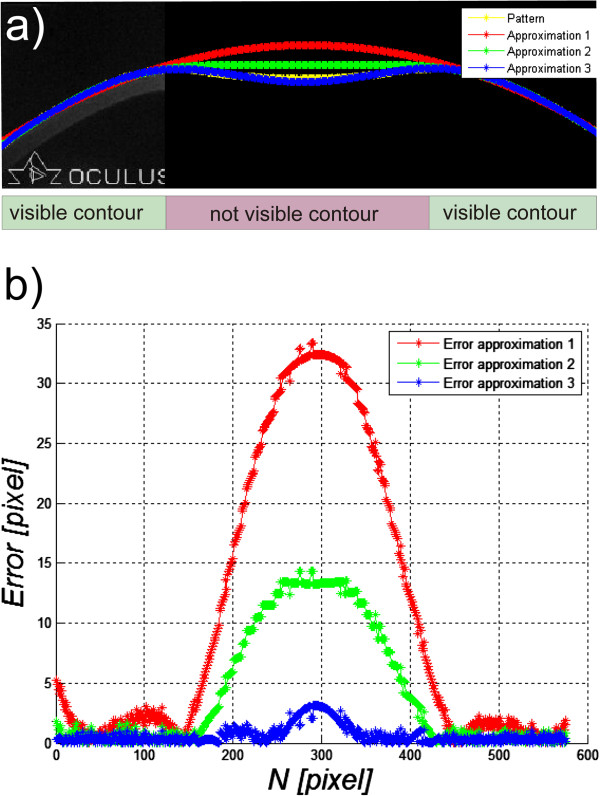
**Images of the section of deflected cornea in the Corvis tonometer.** Image **a)** shows the likely outer corneal contours designated automatically - red, green and blue. The position of none of these contours is confirmed with the actual state. These are possible, potential locations which are most likely. The reference corneal contour waveform is highlighted in yellow. The calculated errors between approximations and the reference contour are shown in the graph **b)**.

The last example is detection of the outer corneal contour in the images from the Corvis tonometer [50-54]. In this case, the occurring shadows and uneven lighting brightness result in a lack of continuity in the detected outer corneal contour. Most often, the middle part of the corneal contour is difficult to see. Then it is necessary to manually correct the results in the middle range. The problems occur in the case when the contour is not visible at any stage of image analysis and the operator's task is to identify it manually. Such cases are shown in Figure 14a). The possible waveforms of the outer corneal contour visible in Figure 14a) show the variety of possible behaviours of the cornea. The expert’s knowledge and machine learning cannot be used in this case. Moreover, no information from the image itself and, in particular, from the corneal contour can be used here. Figure 14b) shows the graph of errors for 3 individual sample approximations - lack of visibility of the contour in the middle part of the image. The resulting maximum errors are 33, 14 and 3 pixels and are arranged in the middle of the image. In this case, however, the reference contour waveform is available. In practical cases, it is not possible by definition. Therefore, the error waveform is not known (Figure 14b) and there is no information which curve that approximates the contour is correct. Generally, for the 13’000 collected images, 100 different patients were selected for the time of first applanation with a clearly visible and automatically recognized corneal contour. Then, 212 central columns of the image were removed, leaving the extreme (right and left) 100 columns. Then, the thus modified image was analysed by measuring the measurement error between the approximation of the corneal contour (in the missing part of the image) and the actual known contour waveform - as in Figure 14. For the analysed 100 images, this error did not exceed 47%. The value of this error arises from the difficulty in estimating the phase of the corneal deflection and other phenomena occurring therein on the basis of visible corneal contours.

In summary, in cases where the recorded data are retrospective, the exclusion criterion should be applied. However, the best solution is a direct contact with the operator during measurements and standardization of basic restrictions and assumptions concerning the performed acquisition of images. In cases where the object contour is not visible (even when using advanced methods of image analysis and processing), anthropometric data, an expert system or information from other more accurate devices or methods capable of performing more accurate calculations should be used.

The summary of error values obtained for the proposed algorithms for automatic image analysis and processing is shown in Table 4.

**Table 4:** Summary of the impact of image acquisition on the results obtained from dedicated automatic algorithms for image analysis and processing

**Object of imaging**	**Problems in image processing**	**Measurement problem/error for selected images**
Determination of the extent of proliferation in the regenerating rat liver cells using a microscope	Microscope set in different ways – different brightness, focal length	8% (when measuring the degree of saturation of the colour reaction)
Thyroid ultrasound images	Thyroid lobe area marked manually in different ways	2% (difference between experts in the brightness measurement)
Evaluation of patient’s back temperature distribution using thermographic method	Patients were not told to spread their arms slightly during tests	31% (average measurement error resulting from grid displacement outside the patient's body)
Photogrammetric method for assessing postural defects	Effect of incident angle lighting of the patient	9% one light source, 11% two light sources, 1% four light sources
Evaluation of the correctness of performing ablative and non-ablative treatments using thermal imaging method in cosmetology	No verification of the places of laser operation (triggered manually) and the dependence of the error on the shape of the skin area subjected to treatment	18% for the nose, 10% for the cheeks and 7% for the forehead.
Measurement of the iridocorneal angle in the anterior segment of the eye using a tomograph	Lack of full visibility of the iridocorneal angle	0-20% depending on the invisible degree of pathology and the amount of the invisible area
Evaluation of tooth enamel thickness loss using a tomograph	Fragments of the enamel boundary are invisible	15% (maximum difference in determination of the boundary location between an expert and an automatic method – 50 pixels)
Tomographic images of the eye fundus - evaluation of layer thickness	Fragments of the retina boundary are invisible	5% (for such percentage of images not all the layers were fully detected- after the automatic correction)
Calculation of the eye anterior chamber surface area using a tomograph	Difficulties in correct approximation of the invisible lens with a straight line	14% (for the location change of the line approximating the lens in the range of ±8 pixels)
Evaluation of the mechanical properties of the cornea and intraocular pressure in Corvis device	Fragments of the corneal outer contour boundaries are invisible	47% (due to difficulties in assessing dynamic behaviour of the corneal contour)

## Summary

The paper presents the problem of the impact of data acquisition on the results obtained from image analysis and processing and, in particular, the results obtained with dedicated algorithms working fully automatically. The examples described in the previous sections show that in the design of this type of algorithms, particular attention should be paid to the following elements:

○ When images are not acquired retrospectively, their acquisition should be carried out in the most reproducible manner. This applies both to the data acquisition device settings and the patient’s orientation. Even small details not directly related to the patient and visible in the image can significantly complicate the construction of the algorithm. For example, additional items appear on the scene, such as items of clothing that should be automatically removed. Similarly, changing the image resolution affects the change in the sizes of individual filter masks or structural elements in the case of performing morphological operations.

○ In the case of images acquired retrospectively, the exclusion criterion should be used, mainly for images that do not have complete data - objects that are not fully visible on the stage or obscured by other objects. Moreover, the possibility of automatic detection of this type of situations (incomplete visibility of an object) and an appropriate algorithm response to this type of situations should be considered here. The initial construction of the algorithm should be started for a group of data obtained for similar acquisition conditions.

○ Evaluation of the algorithm sensitivity to the change of parameters should be performed taking into account both changes in the parameters of the algorithm itself (binarization thresholds, filter mask sizes, etc.) and the possible acquisition device settings (different image resolution, method of illumination, etc.). This assessment is particularly important when using a practical algorithm, in particular, when evaluating the capacity of analysing data from a different image acquisition device or in another institution or by another operator.

○ In each case, the largest possible number of images should be used. Testing the algorithm for a large number of data reduces its sensitivity and specificity but prevents data overfitting.

○ In the absence of complete information in the image (e.g. invisible contour of an object) anthropometric data, information from other cases (machine learning) or information from previous sections, for example, B-scans of the same object (if they exist) must be used.

○ Each algorithm should be designed taking into account the medical evidence. It includes, for example, the size of recognizable objects which enables to select appropriately the filter mask size or the range of brightness changes that enables to choose binarization thresholds.

These elements should be taken into account while designing algorithms. They enable to determine their versatility and correctness of their operation in other medical institutions. The above examples do not fully cover this important and interesting subject. They are only to signal the problems in the design of automatic algorithms for image analysis and processing and expert systems based on them.

## Abbreviations

AUC: Area under the curve; INL: Inner nuclear layer; IS/OS: Inner segment/outer segment junction; NFL: Nerve fiber layer; ROC: Receiver operating characteristics; ROI: Region of interest; RPE: Retinal pigment epithelium; SVM: Super vector machine.

## Competing interests

The author declares that he has no competing interests.
